# Frequency of Gastroesophageal Reflux Disease in Obese Patients Presenting to Lady Reading Hospital in Peshawar, Pakistan

**DOI:** 10.7759/cureus.77921

**Published:** 2025-01-24

**Authors:** Dilaram Khan

**Affiliations:** 1 Gastroenterology, Lady Reading Hospital, Peshawar, PAK

**Keywords:** achalasia, body mass index, gastroesophageal reflux disease, obesity, scleroderma

## Abstract

Background: Obesity is a serious health issue that affects millions of people worldwide and is associated with a number of comorbid conditions, including gastroesophageal reflux disease. The prevalence of gastroesophageal reflux disease increases with an increase in body mass index; thus, obese patients are at a high risk of developing gastroesophageal reflux disease. The main aim of this study was to determine the frequency of gastroesophageal reflux disease in patients with obesity.

Methods: This descriptive cross-sectional study was carried out at the Gastroenterology Department of Lady Reading Hospital in Peshawar, Pakistan, from June 1, 2023, to December 31, 2023. All obese patients (body mass index >30 kg/m^2^), aged 18-60 years of either sex, with heartburns with or without regurgitation at least three times weekly were included in the study, while patients with a history of esophageal surgery and those with achalasia, scleroderma, esophageal stricture, and esophageal malignancy were excluded from the study.

Results: Among the total 282 obese patients, the majority (53.2%) were women. The mean age was 42.56 years with a standard deviation of 11.4 years. Most of the patients (168, 59.6%) were in the age range of 41-60 years. Seventy-three (25.8%) participants were having gastroesophageal reflux disease.

Conclusion: Gastroesophageal reflux disease is quite common in obese patients. In particular, obese patients with a sedentary or sitting nature of job, older age, high body mass index, and injudicious use of non-steroidal anti-inflammatory drugs and who smoke are at high risk of developing gastroesophageal reflux disease.

## Introduction

Gastroesophageal reflux disease (GERD) is a multifactorial condition that affects 12.5-20% of the adult population in the Western world. It affects more than 25% of the population in the United States and is on the rise [1]. Obesity is predicted to affect approximately 30% of the adult population in the United States (body mass index (BMI) >30 kg/m^2^) [2]. It is linked to several serious comorbidities, including insulin resistance (diabetes mellitus), hypertension, obstructive sleep apnea, cardiovascular diseases, mechanical arthropathies, GERD, and many malignancies [3]. According to statistics, a 25-year-old obese man has a 12% lower life expectancy than someone of average weight [4].

Obese patients account for more than 50% of all GERD cases [5]. The prevalence of GERD increases with BMI; in patients with a BMI of less than 25 kg/m^2^, the prevalence is 23%, in patients with a BMI of 25-30 kg/m^2^, the prevalence is 25%, and in patients with a BMI of more than 30 kg/m^2^, the prevalence is above 50% [6]. A direct link between BMI and erosive esophagitis has also recently been reported [6]. In 35% of patients, a 5U increase in BMI causes an increase in the incidence of Barrett's esophagus (BE) [5]. Visceral obesity is more relevant than peripheral obesity in this scenario. The incidence of esophageal and heart malignancies has been linked to obesity. Overweight people have a fourfold increased risk of esophageal cancer compared with people with a normal BMI [7]. In Pakistan, a study done by Butt and Hashemy showed that 46.2% of their 692 GERD patients had a high BMI (>25 kg/m^2^) [8].

According to Takahashi et al., the prevalence of GERD in obese patients was 24.2%. They reported that the prevalence of GERD was 20.7% in patients with a BMI of 30-34.9, 24% in those with a BMI of 35-39.9, 25.2% in those with a BMI of 40-44.9, 26.7% in those with a BMI of 45-49.9, and 24.8% in those with a BMI of >50 [9]. Another study conducted by Sakaguchi et al. found that 26.9% of their patients with a BMI of more than 30 kg/m^2^ had GERD [10].

Despite the high frequency of GERD in the general population, the cause remains unknown. Obesity, alcohol use, smoking, consumption of coffee, tea, soft drinks, and non-steroidal anti-inflammatory drugs (NSAIDs), and sleeping position are all significant lifestyle variables linked to GERD. Obesity and GERD are prevalent health problems in the area, but the prevalence of GERD in obese patients remains unknown in the local population. This research lays the foundation for this direction. The findings of this study will be shared with local health officials to update GERD statistics in the area and ultimately future strategies for the management of GERD.

## Materials and methods

Study design

This descriptive cross-sectional study, consisting of 282 patients, was conducted at the Gastroenterology Department of Lady Reading Hospital in Peshawar, Pakistan, from June 1, 2023, to December 31, 2023.

Inclusion criteria

All obese patients with a BMI of >30 kg/m^2^ of either sex in the age range of 18-60 years were included in the study.

Exclusion criteria

Patients with a history of esophageal surgery, achalasia, scleroderma, esophageal stricture, and esophageal malignancy were excluded from the study.

Operational definitions

GERD

It is a primary condition of the lower esophageal sphincter (LES) characterized by heartburn with or without acid regurgitation at least three times per week, without alarm signs such as recurrent dysphagia, unintended weight loss, hematemesis, anemia, or melena.

Obesity

It is defined as a BMI of >30 kg/m^2^.

Sample size

The sample size for this study was 282 obese patients taking the 24.2% prevalence of GERD in obese patients [9]. The sample size was calculated by applying the WHO calculator for sample size determination taking a 95% confidence interval and a 5% margin of error.

Sampling technique

The sampling technique used was the non-probability consecutive sampling.

Diagnostic method

GERD was diagnosed clinically by taking a detailed history and doing a clinical examination.

Ethical consideration

Proper approval was granted by the Institutional Review Board of Lady Reading Hospital (approval number: 343/LRH/MTI) on May 15, 2023.

Data collection procedure

All patients presenting to the Gastroenterology Department of Lady Reading Hospital in Peshawar, Pakistan, who fulfilled the inclusion criteria were assessed. A detailed history of these patients, including history of NSAIDs and smoking, and clinical examination and esophagogastroduodenoscopy (EGD) were considered necessary to rule out any stricture, carcinoma, ulcers in the stomach or duodenum, etc.

All information regarding age, sex, and BMI was recorded on the predesigned proforma.

Data analysis

Data were analyzed using IBM SPSS Statistics for Windows, Version 22.0 (Released 2013; IBM Corp., Armonk, New York, United States). The mean and standard deviation were calculated for numerical variables such as age and BMI. Frequency and percentages were calculated for categorical variables such as sex and GERD. The frequency of GERD was stratified based on age, sex, and BMI to see the effect modification. Post-stratification, the chi-squared test was applied by keeping the p-value of <0.05 as statistically significant. All results are presented in the form of tables.

## Results

There were 132 (46.8%) men and 150 (53.2%) women with a male-to-female ratio of 0.88:1 and a mean age of 42.56±11.4 years. Most of the patients (168, 59.6%) were in the age range of 41-60 years. GERD was found in 73 (25.8%) patients, of whom 33 (45.2%) were men and 40 (54.8%) were women (Table 1).

**Table 1:** Comparison of gender according to gastroesophageal reflux disease (n=282)

Gender	Gastroesophageal reflux disease		P-value
	Yes	No	
Male	33 (45.2%)	99 (47.3%)	0.75
Female	40 (54.8%)	110 (52.7%)	

The majority of the GERD patients, 53 (72.6%), were in the age range of 41-60 years (Table 2).

**Table 2:** Comparison of age according to gastroesophageal reflux disease (n=282)

Age (years)	Gastroesophageal reflux disease		P-value
	Yes	No	
18-40	20 (27.4%)	90 (43.1%)	0.01
41-60	53 (72.6%)	119 (56.9%)	

There were 119 (42.2%) patients with class I obesity, 110 (39.1%) patients with class II obesity, and 53 (18.7%) patients with class III obesity (Table 3).

**Table 3:** Comparison of body mass index according to gender (n=282)

Body mass index	Gender		P-value
	Male	Female	
Class I obesity (30-34.9 kg/m^2^)	59 (20.9%)	60 (21.3%)	0.24
Class II obesity (35-39.9 kg/m^2^)	49 (17.4%)	61 (21.6%)	
Class III obesity (≥40 kg/m^2^)	24 (8.5%)	29 (10.3%)	

In patients with class I obesity, GERD was found in 13 (17.9%) patients, and in patients with class II obesity, 25 (34.6%) patients were found to have GERD, while in class III obesity, 35 (47.5%) patients with GERD were found. This difference was statistically significant (p<0.001) (Table 4).

**Table 4:** Comparison of gastroesophageal reflux disease according to body mass index (n=282)

Body mass index (kg/m^2^)	Gastroesophageal reflux disease		P-value
	Yes	No	
30-34.9	13 (17.9%)	106 (50.7%)	0.001
35-39.9	25 (34.6%)	85 (40.6%)	
≥40	35 (47.5%)	18 (8.7%)	

There were 33 (45.2%) male patients with GERD, while 40 (54.8%) female patients had GERD; this difference was statistically insignificant (p=0.75) (Table 5).

**Table 5:** Comparison of gastroesophageal reflux disease according to gender (n=282)

Gender	Gastroesophageal reflux disease		P-value
	Yes	No	
Male	33 (45.2%)	99 (47.3%)	0.75
Female	40 (54.8%)	110 (52.7%)	

In patients having a history of NSAID use, the frequency of GERD was found in 49 patients (67.1%), while in patients with no history of NSAID intake, 24 patients (32.9%) were found to have GERD. Applying the chi-squared test, this difference was statistically significant (p<0.02) (Table 6).

**Table 6:** Stratification of the frequency of gastroesophageal reflux disease based on NSAIDs (n=282) NSAIDs: non-steroidal anti-inflammatory drugs

Use of NSAIDs	Gastroesophageal reflux disease			P-value
	Yes	No	Total	0.02
NSAID user	49 (67.1%)	111 (53.1%)	160 (100%)	
Non-user	24 (32.9%)	98 (46.9%)	122 (100%)	
Total	73 (25.8%)	209 (74.2%)	282 (100%)	

In patients with smoking history, the frequency of GERD was found in 52 patients (71.2%), while in non-smokers, 21 patients (28.8%) were found to have GERD. On post-stratification, the chi-squared test was applied, and this difference was statistically significant (p<0.001) (Table 7).

**Table 7:** Stratification of the frequency of gastroesophageal reflux disease based on smoking (n=282)

History of smoking	Gastroesophageal reflux disease			P-value
	Yes	No	Total	0.001
Smoker	52 (71.2%)	99 (47.4%)	151 (100%)	
Non-smoker	21 (28.8%)	110 (52.6%)	131 (100%)	
Total	73 (25.8%)	209 (74.2%)	282 (100%)	

## Discussion

Obesity is principally linked to morbidity related to diabetes and cardiovascular diseases. However, there are many gastrointestinal and liver disorders for which obesity is the direct cause (e.g., fatty liver disease) or an important risk factor for GERD and stones in the gallbladder [11]. GERD is a gastric motility disorder and is defined as the reflux of gastric contents into the esophagus that causes discomfort and other symptoms, such as heartburn and/or acid regurgitation, and can also cause injury to the esophageal mucosa if it occurs at least once per week [12]. Recently, GERD has become one of the most common diseases. The increase in the number of patients is strongly linked to a variety of factors such as obesity, lifestyle, occupation, and nutritional habits [13].

In our study, the frequency of GERD in obese patients was 25.7%. These results are comparable to the results of a systematic review conducted by El-Serag et al., where GERD was present in 18.1-27.3% of obese patients [14]. Similarly, our results are nearly similar to the study results of Xie et al., where the frequency of GERD was 30% in obese patients [15].

This study showed that the frequency of GERD was significantly higher (p<0.001) in patients with high BMI (47.5% in those with a BMI of >40, 34.6% in those with a BMI of 35-39.9, and 17.9% in those with a BMI of 30-34.9; p<0.001). Chowdhury et al. also showed in their study that the prevalence of GERD was high in patients with an increased BMI, which is similar to the results of our study [16]. Vaishnav et al. also reported a significant association (p<0.05) of high BMI with GERD, which is consistent with our findings [17].

In the current study, older aged individuals were found to have a high frequency of GERD compared to young individuals (72.6% vs. 27.4%; p<0.01), and the same were the findings of Vaishnav et al. [17].

In this study, smokers had a higher frequency of GERD (72.2% vs. 28.8%; p<0.001). Almourgi et al. reported in their study that smokers were more likely to have a high prevalence of GERD, consistent with the findings of our study [18]. Similarly, in their review, Maret-Ouda et al. documented a relationship between cigarette smoking and GERD, which is comparable with the results of our study [19].

This study also showed that NSAID users were found to have a high frequency of GERD (67.1% vs. 32.9%; p<0.02).

Limitations of the study

This was a single-center study and may not be a true representative of the community, so large multi-center studies are needed to further elaborate this condition in obese patients.

## Conclusions

Obesity is a common medical condition associated with GERD. In obese patients, the frequency of GERD was found to be 25.7% with increasing weight and age. NSAID misuse, smoking habits, and a sedentary or sitting nature of job are highly likely to cause the development of GERD in these patients.

## References

[1] Jirapinyo P, Thompson CC (2021). Endoscopic gastric plication for the treatment of GERD and underlying class I obesity. VideoGIE.

[2] Sawas T, Marya NB, Storm AC, Blackmon SH, Abu Dayyeh BK (2020). Laparoscopic hernia repair and fundoplication with endoscopic sleeve gastroplasty for complex hernia and GERD management in morbid obesity. VideoGIE.

[3] Miron I, Dumitrascu DL (2019). Gastrointestinal motility disorders in obesity. Acta Endocrinol (Buchar).

[4] Palermo M, Serra E, Duza G (2019). N-sleeve gastrectomy: an option for obesity and GERD. Arq Bras Cir Dig.

[5] Quitadamo P, Zenzeri L, Mozzillo E (2018). Gastric emptying time, esophageal pH-impedance parameters, quality of life, and gastrointestinal comorbidity in obese children and adolescents. J Pediatr.

[6] Dağlı Ü, Kalkan İH (2017). The role of lifestyle changes in gastroesophageal reflux diseases treatment. Turk J Gastroenterol.

[7] Eslami O, Shahraki M, Bahari A, Shahraki T (2017). Dietary habits and obesity indices in patients with gastro-esophageal reflux disease: a comparative cross-sectional study. BMC Gastroenterol.

[8] Butt AK, Hashemy I (2014). Risk factors and prescription patterns of gastroesophageal reflux disease: HEAL study in Pakistan. J Pak Med Assoc.

[9] Takahashi K, Seki Y, Kasama K (2020). Prevalence of reflux esophagitis in obese Japanese undergoing bariatric surgery. JGH Open.

[10] Sakaguchi M, Amano M, Takao M, Sasai Y (2016). The correlation between gastroesophageal reflux disease with obesity and lifestyle related diseases. Ther Res.

[11] Camilleri M, Malhi H, Acosta A (2017). Gastrointestinal complications of obesity. Gastroenterology.

[12] Bor S, Lazebnik LB, Kitapcioglu G, Manannikof I, Vasiliev Y (2016). Prevalence of gastroesophageal reflux disease in Moscow. Dis Esophagus.

[13] Baird DC, Harker DJ, Karmes AS (2015). Diagnosis and treatment of gastroesophageal reflux in infants and children. Am Fam Physician.

[14] El-Serag HB, Sweet S, Winchester CC, Dent J (2014). Update on the epidemiology of gastro-oesophageal reflux disease: a systematic review. Gut.

[15] Xie M, Deng L, Fass R, Song G (2024). Obesity is associated with higher prevalence of gastroesophageal reflux disease and reflux related complications: a global healthcare database study. Neurogastroenterol Motil.

[16] Chowdhury SD, George G, Ramakrishna K (2019). Prevalence and factors associated with gastroesophageal reflux disease in southern India: a community-based study. Indian J Gastroenterol.

[17] Vaishnav B, Bamanikar A, Maske P, Reddy A, Dasgupta S (2017). Gastroesophageal reflux disease and its association with body mass index: clinical and endoscopic study. J Clin Diagn Res.

[18] Almourgi MA, Alamri TM, Algashmari AF, Nassir RA, Alharthi AA, Alsharief QF (2022). Prevalence of smokers among gastroesophageal reflux disease patients in Western Saudi Arabia region. Pharmacophore.

[19] Maret-Ouda J, Markar SR, Lagergren J (2020). Gastroesophageal reflux disease: a review. JAMA.

